# The Telluride YAP/TAZ and TEAD Workshop: A Small Meeting with a Big Impact

**DOI:** 10.3390/cancers15194717

**Published:** 2023-09-25

**Authors:** Guy L. Weinberg, Peter Salamon, John M. Lamar

**Affiliations:** 1 Department of Anesthesiology, University of Illinois at Chicago, Chicago, IL 60612, USA; guy@uic.edu; 2 Department of Mathematics and Statistics, San Diego State University, San Diego, CA 92182, USA; psalamon@sdsu.edu; 3 Department of Molecular and Cellular Physiology, Albany Medical College, Albany, NY 12208, USA

Funding the research needed to advance our understanding of rare cancers is very challenging. Given the limited potential for financial reward and lack of public awareness, inspiring government, industry, or society to fund research for individual diseases is extremely difficult. Nevertheless, patients and families affected by rare cancers will often find novel ways to support and pay for the science that is necessary to promote knowledge of underlying diseases and develop more effective treatments. This is the case for epithelioid hemangioendothelioma (EHE), a one-in-a-million vascular sarcoma caused by oncogenic fusion proteins involving YAP or TAZ, two downstream, negative effectors of the Hippo Pathway. In June of 2023, scientists active in Hippo-YAP/TAZ research assembled at a workshop hosted by the Telluride Science Research Center (TSRC; https://telluridescience.org, accessed on 21 September 2023, Now, officially named the Telluride Science and Innovation Center, participants will likely always refer to the organization as the TSRC, and we use this acronym/moniker throughout) and funded in part by the Richard Eaton Foundation, EHE Foundation, and CRAVAT Foundation. The workshop’s goal was not to address EHE per se but to discuss larger questions around Hippo signaling and ways of controlling or regulating YAP/TAZ-TEAD activity. The role of this workshop in advancing EHE research is best understood in the context of the TSRC itself.

The TSRC, originally named the Telluride Summer Research Center, was founded in 1984 as a haven for budding fields to explore early, basic scientific questions. By design, Telluride workshops were meant to provide a very different experience than typical large scientific meetings, and the admittedly difficult goal was to run workshops that fostered active, on-site collaboration. This was to be achieved by featuring presentations that fostered constructive interactions within a small topic-focused audience. The presentations were solicited on promising, unpublished ideas, and the sessions were friendly and discussion-heavy. Interaction and discussion spilled over into afternoon hikes and shared meals. While never formally enforced, this idyllic recipe was successfully followed by the earliest workshops, and their legacy created a strong culture at the TSRC in favor of maintaining such a format. In fact, this trademark feature has spread to some pockets of the broader scientific community in which “Telluride-style” presentations have come to mean “extensive discussions expected”. TSRC workshops succeeded in large part due to the fertile environment provided by the incredible scenery of the Telluride mountains, whose beauty inspires great ideas. The Center has grown to over fifty workshops and 1500 participants per year, with nearly half the participants coming from overseas. The board of directors intervened to keep tighter control over workshop quality, size, and focus area, capping workshops at 35 participants. As the new president of the board, Rob Walker, recently said, “The small size, open exchange of ideas, emphasis on unpublished work, and focus on identifying new challenges is unlike anything you will find anywhere else”.

The TSRC “YAP/TAZ and TEAD: at the crossroads of cancer” workshop began in 2017 and has occurred every year since, including remotely (2020) and partially remotely (2021&2022). So far, it has limited participation each year to 20 people to stay true to the characteristics that have defined the TSRC for nearly 40 years. Namely, a small, topic-focused workshop seeking productive collaboration through interactive presentations both in the meeting room and during walks in the woods. It has had participants from every continent (excluding Antarctica) and at every stage of their career, ranging from MacArthur Grant awardees, Howard Hughes Investigators, and department chairs to graduate students and post-docs. This includes prominent scientists from both academia and industry, many of whom regularly speak at international conferences on Hippo signaling. The workshop has achieved its scientific goal of increasing collaboration and interaction. The initial example was a collection of Hippo-related papers that included manuscripts from several of the workshop participants [1,2,3,4,5,6,7,8,9,10,11,12,13], which was organized and edited by Dr. Yutaka Hata after he attended the workshop.

The ‘real-world’ impact of the TSRC model is well demonstrated by many substantive advances in EHE research since the inaugural “YAP/TAZ and TEAD: at the crossroads of cancer” workshop. These include the first transgenic mouse models of EHE [14,15], the first EHE cell lines [16], advances in our understanding of the mechanisms of cell transformation caused by the TAZ-CAMTA1 and YAP-TFE3 fusion proteins [16,17,18], a consensus report on best practices for EHE treatment [19], and three phase I clinical trials of TEAD inhibitors (NCT04665206, NCT05228015, and NCT04857372). Many of these studies and projects were first discussed at the workshop, and some of the resulting manuscripts involve collaborations that were initiated at the workshop [14,15,16,17].

Notably, these collaborations have also resulted in improved EHE awareness and funding for EHE-focused basic research. This also results from mutually beneficial interactions among workshop participants and supportive foundations and EHE advocacy groups, including the CRAVAT Foundation, the Richard Eaton Foundation, and EHE foundations located in the United States, United Kingdom (England), and Australia. In addition to funding many of the EHE projects mentioned above, these organizations have helped defray workshop costs, allowing promising young researchers to attend. In turn, the foundations have also benefitted. They have drawn on workshop participants to present research at foundation-sponsored conferences, patient outreach events, and fundraising efforts. This is yet another example of how the TSRC model promotes fruitful collaborations that help advance basic science.

However, the purpose of this workshop is not solely to promote interaction and collaboration to advance EHE research; it is to do this across the entire Hippo-YAP/TAZ-TEAD field. This pathway has strong therapeutic promise in cancer and other diseases, and despite substantial progress over the past 3 decades, there is still a great deal we do not understand about the pathway and how it contributes to human disease. This is reflected in the diversity of topics presented at the workshop throughout its first 7 years, including talks focused on: Hippo Pathway signaling; mechano-transduction; structure and targeting of TEADs; transcriptional regulation by YAP/TAZ-TEADs; regulation of YAP and TAZ nuclear/cytoplasmic shuttling; novel YAP/TAZ-TEAD regulators; epigenetics; and crosstalk between the Hippo Pathway and other critical signaling cascades.

Cancer is a major focus of the workshop, and numerous cancers, in addition to EHE have been discussed. As has virtually every aspect of cancer biology. Other diseases, such as fibrosis of the kidney and lung, pulmonary hypertension, and even COVID-19, have also been featured. Stem cells, cell fate decisions, development, and tissue homeostasis are other frequent topics. The model systems and research tools presented are equally diverse and innovative and include in vitro work in cell lines as well as in vivo work in flies, zebrafish, mice, rats, and humans. Many important discoveries in the Hippo-YAP/TAZ-TEAD field were previewed by scientists sitting in a classroom at the Telluride Middle School, and discussed with enthusiasm, not unlike that of a middle schooler learning about the wonder of science and discovery for the first time. While the contribution of the workshop to the field is difficult to quantify, the true indication of its value to researchers in this field is the fact that many return and actively participate each year.

Given the therapeutic relevance of the Hippo-YAP/TAZ pathway, there is potential for competition amongst researchers and companies active in this field. Despite this, the TSRC “YAP/TAZ and TEAD: At the crossroads of cancer” workshop has remained an open and highly interactive oasis that has fostered extensive collaboration, not competition. The character of its core participants, the unique 40-year-old TSRC recipe, and the inspiring setting of Telluride all play big roles in this. In 2024, the workshop will mark its eighth year, and it is fitting that several of the manuscripts in this Special Edition of *Cancers* were authored by researchers who have participated in many of the previous workshops. The recipe in 2024 will remain unchanged. High-level scientists will present novel, unpublished data on the biology of the Hippo Pathway and the potential for targeting YAP, TAZ, and their binding partners, the TEADs. The workshop will continue to promote active collaboration that leads to advances in our understanding of the Hippo-YAP/TAZ-TEAD pathway and, ultimately, to improved outcomes for all cancer patients, including those with EHE.

## Data Availability

No new data was generated in this editorial.
